# Immediate versus secondary DIEP flap breast reconstruction: a multicenter outcome study

**DOI:** 10.1007/s00404-020-05779-w

**Published:** 2020-09-07

**Authors:** L. Prantl, N. Moellhoff, U. von Fritschen, R. E. Giunta, G. Germann, A. Kehrer, D. Lonic, F. Zeman, P. N. Broer, P. I. Heidekrueger

**Affiliations:** 1 Centre of Plastic, Aesthetic, Hand and Reconstructive Surgery, University of Regensburg, Franz-Josef-Strauß-Allee 11, 93053 Regensburg, Germany; 2 Division of Hand, Plastic and Aesthetic Surgery, University Hospital, LMU Munich, Munich, Germany; 3 Department of Plastic and Esthetic Surgery, Hand Surgery, Helios Hospital Emil von Behring, Berlin, Germany; 4 Department of Plastic, Reconstructive, Esthetic and Handsurgery, ETHIANUM Klinik, Heidelberg, Germany; 5 Center for Clinical Studies, University Medical Center Regensburg, Regensburg, Germany; 6 Department of Plastic, Reconstructive, Hand and Burn Surgery, Bogenhausen Academic Teaching Hospital, Munich, Germany

**Keywords:** Breast reconstruction, DIEP flap, Immediate breast reconstruction, Delayed breast reconstruction, Microsurgery

## Abstract

**Purpose:**

Immediate breast reconstruction (IBR) at the time of mastectomy is gaining popularity, as studies show no negative impact on recurrence or patient survival, but better aesthetic outcome, less psychological distress and lower treatment costs. Using the largest database available in Europe, the presented study compared outcomes and complications of IBR vs. delayed breast reconstruction (DBR).

**Methods:**

3926 female patients underwent 4577 free DIEP-flap breast reconstructions after malignancies in 22 different German breast cancer centers. The cases were divided into two groups according to the time of reconstruction: an IBR and a DBR group. Surgical complications were accounted for and the groups were then compared.

**Results:**

Overall, the rate of partial-(1.0 versus 1.2 percent of cases; *p* = 0.706) and total flap loss (2.3 versus 1.9 percent of cases; *p* = 0.516) showed no significant difference between the groups. The rate of revision surgery was slightly, but significantly lower in the IBR group (7.7 versus 9.8 percent; *p* = 0.039). Postoperative mobilization was commenced significantly earlier in the IBR group (mobilization on postoperative day 1: 82.1 versus 68.7 percent; *p* < 0.001), and concordantly the mean length of hospital stay was significantly shorter (7.3 (SD3.7) versus 8.9 (SD13.0) days; *p* < 0.001).

**Conclusion:**

IBR is feasible and cannot be considered a risk factor for complications or flap outcome. Our results support the current trend towards an increasing number of IBR. Especially in times of economic pressure in health care, the importance of a decrease of hospitalization cannot be overemphasized.

## Introduction

Breast reconstruction is an integral part of holistic breast cancer treatment. It lessens the profound impact that breast cancer diagnosis and therapy have on patients psyche [1] and thus constitutes to an increase of quality of life, social participation and improved self-esteem [2–5].

Several reconstructive approaches exist [6]. Currently, immediate breast reconstruction (IBR) at the time of mastectomy is gaining popularity [7, 8]. Studies show that IBR has no negative impact on recurrence or patient survival, even in patients with advanced disease [9, 10].

For various reasons, implant-based techniques are used for a majority of patients that receive IBR [8, 11]. However, outcome research suggests that autologous tissue transfer shows superior long-term satisfaction, more stable aesthetic results and higher quality of life [12–14]. To this end, the free deep inferior epigastric artery perforator**-**(DIEP) flap has proven to be a working horse for autologous breast reconstruction [15, 16].

Immediate and delayed breast reconstruction (DBR) have been compared with regard to complication rates and clinical outcomes. Reports show better aesthetic outcome, less psychological distress and lower treatment costs following IBR [6, 17, 18]. However, most studies focus on comparing autologous with implant-based reconstructions [6–8, 11], or immediate and delayed autologous reconstructions in women who require post-mastectomy radiation therapy (PMRT) [19, 20]. To date, there is a paucity of significant data on overall complications after immediate versus delayed autologous flap reconstruction. To our knowledge, the largest series compared 910 DIEP flaps in a multicenter retrospective cohort study, including merely 397 cases of IBR [21].

To shed further light onto this topic, we performed a comprehensive analysis of 4577 DIEP free flap breast reconstructions at 22 different German breast cancer centers and compared outcomes and complications after IBR vs. DBR.

## Materials/patients and methods

### Design of the online registry

The German Society of Plastic, Reconstructive and Aesthetic Surgeons (DGPRÄC) initiated a prospective online registry in 2011, to present the structure and quality of free flap breast reconstructions in Germany [22]. Details with regard to the registry and the plastic surgical centers involved have been previously published by Fritschen et al. [22]. In short, plastic surgical centers were certified by the DGPRÄC, before data entry. Ethical approval was obtained from the ethics commission of the Bavarian State Medical Association (156/17 S) and the Berlin Chamber of Physicians (Eth-V-Q/17) prior to initiation of the registry. Data of free flap breast reconstructions were entered intraoperatively, or immediately postoperatively, in a prospective manner and included patients’ demographics, patients’ characteristics, perioperative details, postoperative complications and free flap outcome. Follow-up data of free flap outcome were entered for 3 months postoperatively in the database. Centers were audited and monitored with regard to the quality and stringency of the data entered in comparison with the hospital’s internal documentation [22]. Parts of this database have been previously evaluated by our study group [23].

### Collection of data

22 centers performed DIEP flap reconstructions between January 2011 and January 2019. Data of a total of 3926 female patients, that underwent 4577 free DIEP-flap breast reconstructions, were included in this study. The study included only women that received uni- or bilateral DIEP flap breast reconstruction due to breast cancer. 629 Patients received simultaneous bilateral DIEP-flap reconstruction. If contralateral reconstruction was achieved using a different flap type, we only included the DIEP flap in the study. “Salvage” DIEP flaps performed for women with complications after reconstruction using other free flaps or implant-based reconstructions (e.g., infection, extrusion, or severe pain/cosmetic failure caused by capsule formation) were also included in this study. To analyze a uniform group of patients, women that received breast reconstruction using a different reconstructive approach (i.e., muscle-sparing transverse rectus abdominis-(msTRAM)) were excluded from the study. Other than that, there were no distinct exclusion criteria. However, a complete dataset for every patient to be included was mandatory. The completeness of inclusion was verified by an auditing team. The cases were divided into two groups according to the time of reconstruction: an IBR and a DBR group. Surgical complications were accounted for and the groups were then compared. Clinical outcome analysis was performed by plastic surgeons at the individual centers. The outcomes investigated included total flap loss, partial flap loss, unexpected or emergent revision surgery and reasons for revision surgery (arterial and venous thrombosis, surgical site infection, hematoma donor or recipient site), wound healing disturbances and any medical complications (i.e., deep vein thrombosis, pulmonary embolism, myocardial infarct and others) that occurred postoperatively.

### Statistical analysis

Sample size: No a priori sample size calculation was performed for this study for two reasons. The expected number of patients in the chosen time interval was high enough for detection of any clinically relevant difference without having the risk of being under powered. There was no primary endpoint for a sample size calculation, since this is an exploratory trial with several different endpoints.

Statistical methods: Data are shown as mean (standard deviation) or as absolute and relative frequencies. A *t*-test or a Chi squared test of independence was used to determine differences between groups. Results were considered statistically significant at a probability level of ≤ 0.05 to guide conclusions. All analyses were performed using SAS (Version 9.4, The SAS institute, Cary, NC).

## Results

### Demographics and patient characteristics

Patients´ characteristics are shown in Table 1. The IBR group included 897 patients (1136 free flaps, mean age 49.9 years (SD 11.5)) and the DBR group included 3016 patients (3441 free flaps, mean age 51.8 years (SD 35.8)).

**Table 1:** Patient characteristics

Patient characteristics	Immediate reconstruction	Delayed reconstruction	*p* value
Patients, *n*	897	3016	
Free flaps, *n*	1136	3441	
Age, years			
Mean (SD)	49.9 (11.5)	51.8 (35.8)	0.082
BMI, kg/m^2^			
Mean (SD)	26.2 (4.8)	26.3 (4.3)	0.733
Smoking history, *n* (%)	106 (9.3)	370 (10.8)	0.192
Comorbidities, *n* (%)			
Diabetes mellitus	32 (2.8)	93 (2.7)	0.921
Coagulopathy^a^	15 (1.3)	56 (1.6)	0.557
Abdominal scar > 10 cm, *n* (%)	27 (2.4)	165 (4.8)	0.001
Family history of breast and/or ovarian cancer in FDRs, *n* (%)	346 (30.5)	845 (24.6)	< 0.001
Genetic disposition, *n* (%)^b^	254 (22.4)	443 (12.9)	< 0.001
Chemotherapy within last 6 months, *n* (%)^c^	426 (37.5)	2179 (63.3)	< 0.001
Chemotherapy later than 6 months, *n* (%)^d^	243 (21.4)	1963 (57.0)	< 0.001
Immunosuppressive therapy, *n* (%)^e^	6 (0.5)	28 (0.8)	0.44
Tamoxifen therapy, *n* (%)^f^	101 (8.9)	383 (11.1)	0.038
Neoadjuvant radiation therapy, *n* (%)	209 (18.5)	1433 (41.6)	< 0.001
Indication, *n* (%)			< 0.001
Status after mastectomy	22 (2.0)	1533 (56.3)	
DCIS	139 (12.4)	41 (1.5)	
Primary carcinoma	381 (34.0)	55 (2.0)	
Familial risk^g^	223 (19.9)	39 (1.4)	
Complications after other reconstruction^h^	12 (1.1)	801 (29.4)	
Benign tumor	26 (2.3)	21 (0.8)	
Status after BCT	193 (17.2)	128 (4.7)	
Tumor recurrence	92 (8.2)	30 (1.1)	
Other	31 (2.8)	74 (2.7)	

Comparison of patients´ demographics, comorbidities, risk factors for breast cancer, systemic breast cancer treatment and reasons (indication) for DIEP flap reconstruction. Percentages for each item were calculated based on the number of free flaps in each group

n, number; SD, standard deviation; BMI, Body Mass Index; FDR, first degree relatives; BCT, Breast-Conserving Therapy; DCIS, Ductal Carcinoma in Situ

^a^Self-reported clinical history of any derangement of hemostasis resulting in impaired clot formation

^b^No positive test but a positive family history for breast cancer

^c^Neoadjuvant Chemotherapy within the last 6 months prior to breast reconstruction

^d^Neoadjuvant Chemotherapy more than 6 months prior to breast reconstruction

^e^Immunotherapy using targeted antibodies for tumors that overexpress HER-2 protein receptor

^f^Patients with hormone-receptor positive breast cancer receiving tamoxifen therapy

^g^i.e., risk-reducing mastectomy due to pathogenic mutation identified in genetic test for familial/hereditary breast cancer

^h^Complications after previous reconstructive procedure (i.e., implant reconstruction)

Preoperative evaluation revealed no significant differences regarding perioperative risk factors such as BMI, comorbidities (diabetes mellitus, coagulopathy), or smoking history (*p* > 0.05). Merely, a significantly higher prevalence of abdominal scars was observed (4.8 percent versus 2.4 percent; *p* = 0.001) in the DBR group.

Risk factors for breast cancer, such as a positive family history of breast and/or ovarian cancer in first degree relatives (FDRs) (30.5 versus 24.6 percent of cases; *p* < 0.001) and genetic disposition (22.4 versus 12.9 percent of cases; *p* < 0.001) were significantly higher in the IBR group.

With regard to systemic breast cancer therapy, neoadjuvant chemotherapy was administered significantly more frequently in the IBR group (within 6 months prior to reconstruction: 37.5 versus 63.3 percent of cases, *p* < 0.001; more than 6 months prior to reconstruction: 21.4 versus 57.0 percent of cases, *p* < 0.001). Conversely, patients in the DBR group received Tamoxifen therapy significantly more frequently (8.9 versus 11.1 percent of cases; *p* = 0.038). Immunotherapy using targeted antibodies was comparable between both groups (*p* > 0.05). A significantly lower number of patients in the IBR group received neoadjuvant radiation therapy (18.5 versus 41.6 percent of cases; *p* < 0.001).

The indication for DIEP flap reconstructions differed significantly between the groups (*p* < 0.001). Indications for patients in the IBR group predominantly included primary carcinoma (34.0 percent), familial risk (19.9 percent), or ductal carcinoma in situ (DCIS, 12.4 percent), whereas most patients of the DBR group required reconstruction after mastectomy (56.3 percent) or due to a complication after an unsuccessful other type of breast reconstruction (29.4 percent).

### Perioperative details and postoperative complications

All perioperative characteristics are summarized in Table 2. Mean ischemia time was significantly shorter in the IBR group (44 (SD26.1) versus 53 min (SD25.4); *p* < 0.001), whereas mean operative time did not differ significantly (315 (SD144.6) versus 320 (SD121.9) minutes; *p* = 0.29).

**Table 2:** Perioperative details according to timing of reconstruction

Perioperative details	Immediate reconstruction	Delayed reconstruction	*p* value
Free flaps, *n*	1136	3441	
Reconstructed side, *n* (%)			< 0.001
Right	322 (28.3)	1238 (36.0)	
Left	344 (30.3)	1332 (38.7)	
Both	470 (41.4)	871 (25.3)	
Operation time, min			
Mean (SD)	315 (144.6)	320 (121.9)	0.29
Ischemia time, min			
Mean (SD)	44 (26.1)	53 (25.4)	< 0.001
Recipient vessels, *n* (%)			
Internal mammary	958 (84.3)	2725 (79.2)	0.002
Thoracodorsal	142 (12.5)	562 (16.3)	0.002
Other	36 (3.2)	154 (4.5)	0.056
Flap monitoring, *n* (%)			
Clinically	1119 (98.5)	3409 (99.1)	0.149
Transcutaneous doppler probe	219 (19.3)	1827 (53.1)	< 0.001
Perivascular doppler probe (i.e., cook)	0 (0.0)	26 (0.8)	0.007
Transcutaneous HbO2 test (i.e., O2C)	0 (0.0)	10 (0.3)	0.146
Warm touch preoperatively	76 (6.7)	146 (4.2)	0.001
Warm touch postoperatively	719 (63.3)	1644 (47.8)	< 0.001
Postoperative mobilization, *n* (%)			
Postop day 1	933 (82.1)	2360 (68.7)	< 0.001
Postop day 2	95 (8.4)	678 (19.7)	
Postop day 3	21 (1.8)	105 (3.1)	
Postop day 4	58 (5.1)	108 (3.1)	
Postop day 5	17 (1.5)	81 (2.4)	
Postop day 6	11 (1.0)	61 (1.8)	
Postop day 7	1 (0.1)	41 (1.2)	
Hospital stay, days			
Mean (SD)	7.3 (3.7)	8.9 (13.0)	< 0.001

Percentages for each item are calculated based on the number of free flaps in each group

n, number; SD, standard deviation; min, minutes

Mean length of hospital stay was significantly shorter in the IBR group (7.3 (SD3.7) versus 8.9 (SD13.0) days; *p* < 0.001). Postoperative mobilization started significantly earlier in the IBR group (mobilization on postoperative day 1: 82.1 versus 68.7 percent; *p* < 0.001).

Postoperative complications are displayed in Table 3. Overall, there was no significant difference between the groups of patients regarding the rate of partial (1.0 versus 1.2 percent of cases; *p* = 0.706) and total flap loss (2.3 versus 1.9 percent of cases; *p* = 0.516). Revision rates were slightly, but significantly lower in the IBR group (7.7 versus 9.8 percent; *p* = 0.039). When analyzing reasons for revision surgery, we found that the rate of hematomas at the recipient site was significantly lower in the IBR group (2.2 versus 3.6 percent; *p* = 0.03).

**Table 3:** Postoperative complications over a follow-up period of 3 months

Postoperative complications	Immediate reconstruction	Delayed reconstruction	*p* value
Free flaps, *n*	1136	3441	
Total flap loss (*n*)	26 (2.3)	66 (1.9)	0.516
Partial flap loss (*n*)	11 (1.0)	40 (1.2)	0.706
Revision surgery, *n* (%)	88 (7.7)	337 (9.8)	0.039
Venous thrombosis	38 (3.3)	85 (2.5)	0.14
Arterial thrombosis	14 (1.2)	60 (1.7)	0.294
Infection donor site	2 (0.2)	21 (0.6)	0.12
Infection recipient site	2 (0.2)	18 (0.5)	0.201
Hematoma donor site	7 (0.6)	30 (0.9)	0.52
Hematoma recipient site	25 (2.2)	123 (3.6)	0.03
Wound-healing disturbances requiring revision surgery, *n* (%)			
Donor site	13 (1.1)	67 (1.9)	0.097
Recipient site	12 (1.1)	58 (1.7)	0.174
Medical complications, *n* (%)	75 (6.6)	219 (6.4)	0.777

Percentages for each item are calculated based on the number of free flaps in each group

n, number

The prevalence of medical complications was comparable between both groups (6.6 versus 6.4 percent; *p* = 0.777) (Table 3).

## Discussion

Immediate breast reconstruction has become the preferred reconstructive approach for women with breast cancer. According to the literature, more than 70% of breast reconstructions are performed at the time of mastectomy [6], for several reasons. Studies have shown that IBR is oncologically safe [24–26]. The introduction of skin-sparing and nipple-sparing mastectomy has allowed immediate breast reconstruction to gain momentum [27]. Here, the native breast skin envelope remains intact, which facilitates good aesthetic results, especially for microvascular autologous tissue-based reconstructions [28–30]. Psychologically, patients receiving IBR show less anxiety or depression and higher levels of self-esteem, sexuality and an increased quality of life [4, 31, 32].

As stated previously, most IBRs are implant-based reconstructions [8, 11, 27]. Several studies provide an explanation for this phenomenon. Exemplary, while physician payments for prosthetic reconstruction have risen over the past years, there is a stagnation in compensation for autologous reconstruction. In addition, there is a significant opportunity cost related to autologous breast reconstruction due to high physician effort, long operative hours and extended hospitalization [33–35]. Nevertheless, autologous tissue transfer shows superior long-term results [12–14]. Prior studies have compared overall outcome and complications of immediate and delayed breast reconstructions, mostly, however, without differentiating between autologous and prosthetic reconstruction and producing inconsistent conclusions with regard to an increase of complications in either group [6, 36–39].

Hence, this study aimed at generating a distinguished analysis for DIEP-flaps, the commonly preferred approach for autologous breast reconstruction [15, 16]. Based on the largest patient series to date, the presented study compared the outcomes of 4577 DIEP free flap reconstructions with regard to the timing of reconstruction. Overall, we found no significant difference in the rate of total or partial flap loss when comparing IBR with DBR. In this regard, our data agree with a recent study of Beugels et al. who also describe no significant differences in major complications or flap re-explorations between immediate and delayed reconstructions [21]. Conversely, they found significantly more hematomas in immediate reconstructions and a higher rate of wound healing disturbances in delayed reconstructions [21], which is not supported by our data. Our data show a slightly, but significantly higher rate of revision surgery in DBRs which can be attributed to a significantly higher rate of hematomas at the recipient site in this group. Operating in scarred and fibrous tissue, as is the case in many DBR patients that received neoadjuvant radiation and mastectomy, may cause higher rates of hematoma and thus also higher surgical revision rates [40].

According to Fischer et al. autologous IBR adds a significant risk for venous thromboembolism, including deep venous thrombosis and pulmonary embolism [37]. Contrary, in our investigated group of DIEP-flap reconstructions, this finding did not hold true. Instead, our results show that postoperative medical complications were comparable between both groups and rates of venous flap thrombosis were similar.

While mean ischemia time was longer in the DBR group, operative times in total did not differ between both groups. This can be attributed to the standard two team approach often utilized for autologous tissue reconstructions.

From an economic point of view, it is widely agreed upon that IBR is more cost-effective, as opposed to DBR [17, 41]. IBR involves less surgical procedures, thus recovery periods and hospitalization are reduced. In line with this, our data show significantly reduced hospitalization after IBR, as compared to DBR. Also, we found significantly earlier postoperative mobilization in the IBR group. In several surgical disciplines, early mobilization has been associated with a decrease of hospitalization, and thus health-related costs [42–44]. While the exact reasons for earlier mobilization in the IBR group remain unclear, these findings should further encourage IBR for the benefit of the patient, but also with regard to reducing health economic costs in times of rising economic pressure in healthcare. Despite being more cost-effective, in our study, merely ~ 25% of all DIEP reconstructions were performed immediately at the time of mastectomy. While only a hypothesis, we think this could be explained by the comparatively low reimbursement associated with IBR in Germany, ranging between ~ 13.500 € for unilateral and ~ 18.500 € for bilateral reconstruction [45]. However, a two-staged approach, i.e., a separation of mastectomy and reconstruction into two operative procedures and hospital stays, increases physicians´ revenue.

The two groups were comparable with regard to age, mean BMI, the prevalence of comorbidities such as diabetes mellitus and coagulopathy, as well as nicotine abuse and immunosuppressive therapy prior to reconstruction. We did, however, observe significant differences with regard to chemotherapy and tamoxifen administration between both groups. Chemotherapeutic agents are cytotoxic and are thus considered to affect surgical outcome in breast reconstructions [46–48]. Perioperative tamoxifen therapy may increase the risk of thrombotic flap complications and flap loss [46, 49]. Our results show that patients in the IBR group received neoadjuvant chemotherapy significantly less frequently prior to reconstruction. In addition, although the difference was only marginal, patients in the IBR group received significantly less perioperative tamoxifen therapy. Thus, one may argue that patients in the DBR group were at significantly higher risk to develop postoperative complications, as compared to the IBR group. However, in patients that undergo mastectomy and IBR, recent studies found no increase in morbidity or complication rate with regard to neoadjuvant chemotherapy [47, 48]. Hence, one could interpret that the data points towards surgeons´ prevailing reluctance to perform IBR after chemotherapy, although this may be unfounded.

In our study, we found significantly higher rates of bilateral breast reconstruction in the IBR group. Concordantly, this group showed higher rates of familial risk (defined as a pathogenic mutation identified in genetic test for familial/hereditary breast cancer), higher rates of a positive family history for breast and/or ovarian cancer in FDRs, as well as a significantly higher genetic disposition for breast cancer. An increase in breast reconstructions has been described for patients desiring prophylactic mastectomies secondary to specific genetic mutations [11]. In accordance, the higher number of bilateral breast reconstructions in the IBR group could be related to a higher number of prophylactic contralateral mastectomies in conjunction with breast reconstruction in this group.

A significantly lower number of patients in the IBR group received neoadjuvant radiation therapy, as compared to the DBR group. Currently, the role of neoadjuvant radiation therapy and IBR is discussed as a feasible alternative as opposed to more traditional treatment concepts with a multimodal approach of chemotherapy, surgery, and postmastectomy radiotherapy [50, 51]. Ideal timing of breast reconstruction after radiation therapy is, however, controversially debated and further studies are needed in this regard [52–54].

A major strength of this study lies within the large sample size of 3926 female patients and 4577 DIEP flap breast reconstructions after malignancies in 22 different breast cancer centers. Patients were divided into two groups according to timing of reconstruction. We thus compared 1136 flaps for IBR with 3441 delayed free flaps. This series allows us to draw significant conclusions with regard to outcome and complications of DIEP tissue transfers for breast reconstruction depending on timing of reconstruction, and to our knowledge no previous study analyzed a series as large in this specific patient group.

Our data show significant differences within postoperative monitoring standards between both groups which arguably is a limiting factor of the study. This can be attributed to the multicenter nature of the study, since national standard operating procedures for free flap monitoring remain to be defined. Also, the indication for DIEP flap reconstructions differed between the groups. Unsurprisingly, the main reason for delayed DIEP reconstruction was status after mastectomy, while the primary reason for IBR was primary carcinoma. The inhomogeneity of both groups with regard to neoadjuvant chemo- and radiation therapy must also be considered a limitation of the study. Future studies must conduct multivariate analysis to account for confounding factors within the investigated groups.

## Conclusion

The presented study analyzed the largest series of microsurgical DIEP breast reconstructions in Europe, with regard to the timing of reconstruction. The data show that IBR is feasible and cannot be considered a risk factor for complications or flap outcome. Our results support the current trend towards increasing numbers of IBR. Especially in times of economic pressure in health care, the decrease in hospitalization associated with IBR cannot be overemphasized.
